# Chronic Erosive Dermatopathy (CED) Associated with Ectoparasite Infestation in Nile Tilapia Post-Larvae (*Oreochromis niloticus* Linnaeus, 1758)

**DOI:** 10.3390/vetsci13101052

**Published:** 2026-10-08

**Authors:** Pornpawit Tanpichai, Onanong Charoenwai, Sayamon Srisuwatanasagul, Patharapol Piamsomboon

**Affiliations:** 1 Aquatic Animals Clinic, Faculty of Veterinary Medicine, Mahanakorn University of Technology, Bangkok 10530, Thailand; pornpawit@mut.ac.th; 2 Graduate Program in Veterinary Science and Technology (VST) International Program, Faculty of Veterinary Science, Chulalongkorn University, Bangkok 10330, Thailand; 6578305731@student.chula.ac.th; 3 Department of Anatomy, Faculty of Veterinary Science, Chulalongkorn University, Bangkok 10330, Thailand; sayamon.s@chula.ac.th; 4 Department of Veterinary Medicine, Faculty of Veterinary Science, Chulalongkorn University, Bangkok 10330, Thailand

**Keywords:** aquaculture, erosion, fluke, lateral line canal, parasite, ulcer

## Abstract

Chronic erosive dermatopathy (CED) associated with ectoparasites was investigated in Nile tilapia (*Oreochromis niloticus*). Between 2023 and 2024, craniofacial lesions occurred at a prevalence of <1% across culture stages. Histopathology showed epidermal goblet cell proliferation and disappearance of the muscular layer, with no active pathogens or inflammation. Clinical history indicated heavy ectoparasite infestations during the 0.1 g fry stage, supported by parasite remnants found in deep skin scrapes of CED lesions. A clinical trial assessed this link using 3000 post-larvae (0.01 g each), divided into a control group (untreated) and a treatment group (tank water disinfected with 75% chlorine powder to 15 ppm for 24 h before stocking). Sampling 30 fish weekly for three weeks revealed CED incidence rates of 30% (9/30), 40% (12/30), and 27% (8/30) in controls, whereas the treatment group developed no lesions. The odds of developing CED were 25.7-, 40.0-, and 21.8-fold higher in controls at weeks 1, 2, and 3, respectively. These findings suggest early-stage ectoparasite infestations contribute to CED development. Implementing strict sanitary measures from post-larval stages is essential to minimize prevalence.

## Simple Summary

Chronic erosive dermatopathy (CED) is a skin condition in Nile tilapia that causes facial ulcers. Although fish with CED survive to market size without high mortality, the resulting physical deformities reduce their market value. Investigations revealed that heavy ectoparasite infestations during the early fry stage trigger these lesions, as parasite remnants were found in deep skin scrapes of affected tissue. To confirm this link, a farm trial demonstrated that disinfecting culture tanks and water with chlorine powder before stocking post-larvae completely prevented CED development. In contrast, untreated control groups experienced up to a 40% lesion incidence. These findings highlight that early-stage ectoparasite prevention and strict farm hygiene are essential for protecting fish appearance and maintaining economic value in tilapia farming.

## 1. Introduction

In Thailand, Nile tilapia (*Oreochromis niloticus*) holds the top spot among freshwater aquaculture species, contributing 64.09% to the total production and generating a market value of 36.9 million USD [1]. Moreover, the fish has seen a remarkable surge in global production, increasing from 1.0 million tons to 4.4 million tons between 2000 and 2020 [2]. By 2024, it ranked fourth globally in inland finfish aquaculture, accounting for 11% of total production [3]. Tilapias belong to the order Cichliformes and the family Cichlidae [4]. This family also includes ornamental species, such as Oscars (*Astronotus ocellatus*) and angelfish (*Pterophyllum scalare*), which have been reported to develop chronic erosive dermatopathy (CED), a lateral line disorder [5,6]. 

The fish lateral line, often described as a ‘touch-at-a-distance’ sense, is a mechanoreceptive sensory organ that detects water movement, vibrations, and pressure changes in the surrounding environment [7,8]. The smallest functional unit of the lateral line is the neuromast, a sensory structure consisting of a hair cell epithelium and a cupula that connects the ciliary bundles of the hair cells to the surrounding water [7,9]. These neuromast receptor organs are located on the skin, within bony canals on the head, and in tubed scales along the trunk [10]. Neuromasts located on the skin surface, referred to as superficial neuromasts, are used to detect the direction and speed of water flow. In contrast, canal neuromasts (located within subdermal channels) act as pressure gradient detectors, sensing differences in water movement between adjacent canal pores, primarily caused by turbulence [11]. Focusing on the head region, canal neuromasts are found in the mandibular and orbital regions [12], which represent key areas of interest in our study.

CED is a chronic pathological disorder that affects the lateral line canals of the head and the trunk of various marine and freshwater fish species such as Murray cod (*Maccullochella peelii peelii*) [13,14], Sharpsnout seabream (*Diplodus puntazzo*) [15], European seabass (*Dicentrarchus labrax*) [16], Palette surgeonfish (*Paracanthurus hepatus*) [17], angelfish [6], Oscar [18], and Nile tilapia [19]. Moreover, a previous study in culture meagre (*Argyrosomus regius*) also showed that CED can be occurred since the fish were 28 days post-hatching, which indicated the susceptibility of post-larvae to this disorder [20].

Similar symptoms have been recorded in several fish species as chronic ulcerative dermatopathy (CUD), hole-in-the-head (HIH), head and lateral line erosion syndrome (HLLE), and lateral line depigmentation (LLD) [16,21]. The symptoms manifest as focal erosion, ulceration, and the loss of epidermis around the lateral line canals of the head and trunk. While the definitive cause remains unknown, several factors have been suspected, including groundwater issues in Murray cod [13], high stocking density in Nile tilapia [19], nutritional imbalance in discus fish (*Symphysodon* spp.) [22], and the presence of the protozoan *Spironucleus vortens* in the head lesions of angelfish [6]. The treatment of CED is also constrained by the fact that the causative agent is still unverified. Nevertheless, a study in Palette surgeonfish showed lesion improvement following the use of an opioid antagonist [17], and a study in climbing perch (*Anabas testudineus*) presented the efficacy of neem (*Azadirachta indica*) leaf powder in hole-in-the-head disease [23].

One of the major causes of economic losses in tilapia farming, particularly during the fingerling and juvenile stages, is ectoparasite infestation. These parasites induce a range of health issues in fish, resulting in reduced growth, compromised immunity, increased susceptibility to secondary infections, and high mortality rates [24]. Trichodiniasis, caused by *Trichodina* spp., is a disease triggered by peritrichous ciliated protozoans that infect various aquatic species, including Nile tilapia [25], Common carp (*Cyprinus carpio*) [26], and Cachama (*Piaractus brachypomus*) [27]. These protozoans are typically found in diverse environments where they function as commensals, feeding on external cellular debris; however, pathogenesis occurs when their adhesive discs attach to the epithelial cells, causing tissue irritation [28]. Furthermore, common monogeneans affecting tilapia include gill flukes (*Dactylogyrus* spp.) and skin flukes (*Gyrodactylus* spp.) [29]. These flukes attach to the host using hooks on their opisthaptor, leading to mechanical tissue damage [30]. Infected fish typically display clinical signs such as skin redness, flashing, rapid respiration, and lethargy [31].

In this study, CED was found in Nile tilapia across multiple life stages, from post-larvae to grown-out fish. Although clinical observations by local farmers beside earthen ponds indicated low morbidity (under 1%) and no mortality, the condition negatively affected carcass market value, resulting in substantial economic losses, particularly among grown-out stock [32]. Thus, identifying the etiology and implementing effective management strategies are critical for sustainable tilapia aquaculture. Initial investigation of CED in post-larval tilapia revealed the presence of common ectoparasites in tilapia, including *Trichodina* spp. and monogenean flukes. Based on these findings, a clinical trial was subsequently conducted to test the association between ectoparasite infestation and CED by comparing fish reared in conditions with and without ectoparasite treatment.

## 2. Materials and Methods

### 2.1. History of Clinical Cases

Grow-out Nile tilapia (400–900 g) exhibiting clinical signs of CED were found in November 2023 from a private farm in Kanchanaburi Province, Thailand. Fish were reared in a 1200 m^2^ earthen pond (≈4000 fish; 3.33 fish/m^2^). In July 2024, a similar CED case was identified at a private nursery farm in Nakhon Pathom Province, Thailand, involving Nile tilapia at fry (0.1 g) and fingerling (30 g) stages. Each fish stage was stocked in a separate 4800 m^2^ earthen pond (10,000 fish; 2.08 fish/m^2^). Water quality parameters of these cases were within acceptable ranges, including temperature (28–31 °C), dissolved oxygen (>5 mg/L), pH (6.5–7.5), and total ammonia and nitrite (<0.1 mg/L). Moreover, the water used on each farm came from a natural river basin near the farm. The fish were fed a commercial diet (Thai Union Feedmill, Samut Sakhon, Thailand). The presence of CED was observed at levels below 1% per pond, with no mortality. The resident fisheries professional verified that no antimicrobials or chemicals had been administered to any of the fish for at least two weeks before their submission for disease examination at the Faculty of Veterinary Science, Chulalongkorn University.

### 2.2. Disease Investigation 

During the disease investigation, the fish were anesthetized with chilled water adjusted with an ice pack to examine the skin lesions and perform the ectoparasite examination. Subsequently, the fish were euthanized using an overdose of the anesthetic solution (Aquanes^®^, Better Pharma, Bangkok, Thailand) for further diagnostic methods.

#### 2.2.1. Ectoparasite Examination

Fish weighing 0.1 g were placed on the slide, and tail fin examination using the wet-mounting technique was conducted. For fish weighing more than 30 g, mucus was scraped and placed on the slide. Wet-mounted preparations were analyzed under a light microscope for external parasites. The number of ectoparasites was counted to assess infestation intensity at 10× magnification (10 fields). Infestation levels were classified as follows: no parasites detected, mild infestation (1 parasite per field), moderate infestation (2–5 parasites per field), and heavy infestation (more than 5 parasites per field) [33].

A deep skin scraping was performed at the ulcerated lesion after the fish were euthanized. The fish were placed under a stereoscope, and a sterile scalpel blade was used to scrape the lesion. The collected material was then examined under a light microscope as described above.

#### 2.2.2. Bacterial Culture and PCR Assays 

Bacterial culture and PCR assays were performed to determine whether bacteria or viruses were subclinically present in the affected fish. Firstly, the fish were dissected using aseptic technique; the head kidney was collected, and the tissue was cultured on Tryptic Soy Agar (TSA; Oxoid, Hampshire, UK) and incubated at 25–27 °C overnight. Then, spleen and head kidney were preserved in 70% ethanol and frozen at −20 °C for subsequent PCR [34]. Major infectious diseases in tilapia were investigated, including Tilapia Lake virus (TiLV), Tilapia parvo virus (TiPV), *Streptococcus agalactiae*, *Edwardsiella* spp., *Aeromonas veronii*, and *Mycobacterium* spp. The primers and thermal cycling conditions are provided in Table 1.

#### 2.2.3. Histopathology

The histopathology methodology was adapted from a previous study [42]. The cranium of the fish was dissected and fixed in 4% paraformaldehyde for the standard histopathology procedure. Briefly, the tissues were processed using an automatic tissue processor (Tissue-Tek VIP 5 Jr., Sakura Finetek Japan Co., Ltd., Tokyo, Japan) and embedded in paraffin blocks. The samples were then sectioned into 4 μm thick slices using a microtome (Thermo Fisher Scientific, Waltham, MA, USA) and mounted on slides for Hematoxylin and Eosin staining. Slides were digitized using a slide scanner (3DHISTECH, Budapest, Hungary), and photomicrographs were analyzed with CaseViewer software version 1.4 (3DHISTECH, Budapest, Hungary).

### 2.3. Clinical Trials

Based on the disease investigation described in Section 3, emphasized by ectoparasite infestation in the ulcerative lesions of 0.1 g fish, a clinical trial was conducted to examine the relationship between ectoparasite infestation and the development of CED lesions. Six concrete tanks (1 m × 1.5 m × 2.5 m) were used and divided into 2 groups (triplicate per group). The treatment tank was disinfected with 75% chlorine powder at 15 ppm for 24 h before use. The water column of the treatment group was treated with chlorine and alum prior to filling. In the control group, only the water column was treated with alum to precipitate sediment before stocking. Water quality was monitored and maintained within GAP farm standards including a temperature of 28–31 °C, dissolved oxygen levels >5 mg/L, and a pH range of 6.5–7.5, and total ammonia and nitrite <0.1 mg/L.

The same batch of post-larval tilapia (average body weight 0.01 g) were transferred from a hatchery and stocked into the hapa (0.5 m × 0.5 m × 0.5 m) at 500 fish per hapa inside each concrete tank. Thirty fish (ten fish per hapa) were randomly sampled to confirm the absence of skin ulcers and ectoparasite infestation. Subsequently, 30 fish from each group were randomly sampled weekly to assess skin lesions and ectoparasite infestation using methods described above. In addition, five fish per group per time point were collected for histopathological examination. 

### 2.4. Statistical Analysis

Individual fish from each group were evaluated for the presence (positive) or absence (negative) of skin lesions at each time point. Odds ratios (OR) were calculated using the modified Haldane–Anscombe (mHA) correction [43], and *p*-values were analyzed using Fisher’s exact test to assess differences in the incidence of CED lesions between the two experimental groups. Statistical analyses were performed using SPSS software version 29.0 (SPSS Inc., Chicago, IL, USA).

## 3. Results

### 3.1. Disease Investigation

**Figure 1:**
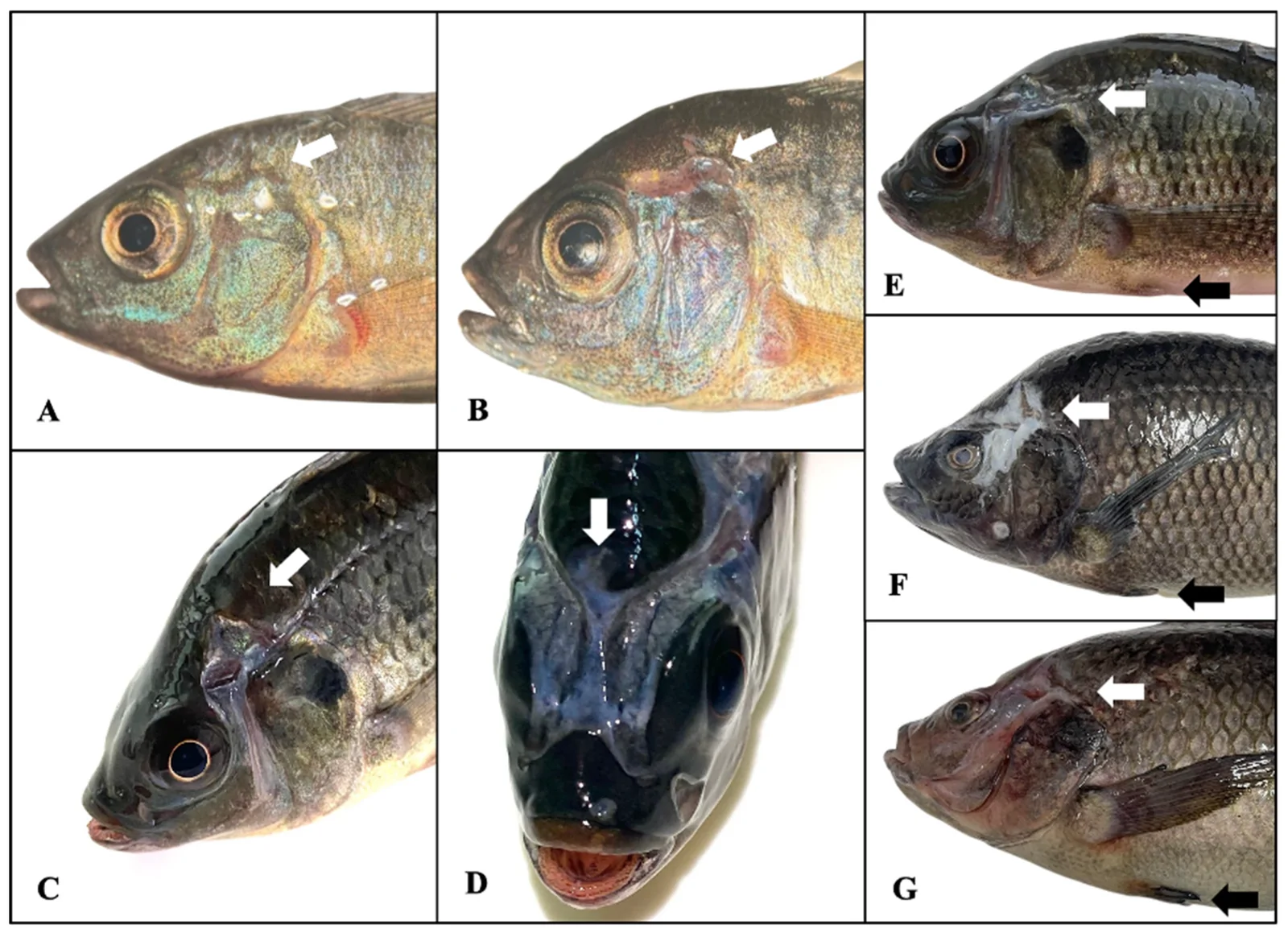
In fish weighing 0.1 g, macroscopic lesions of healthy (**A**) and affected (**B**) tilapia under a stereoscope. The white arrow points to the area of comparison between the healthy and diseased fish. Macroscopic lesions included ulcerative lesions (white arrow) above the operculum, snout, and around the eye, as seen in both lateral (**C**) and craniocaudal (**D**) views, in fish weighing 30 g (**E**), 400 g (**F**), and 900 g (**G**). The black arrow displayed caudal fin erosion.

According to the stereoscopic examination in fish weighing 0.1 g, healthy fish showed intact skin with normal mucus and epidermis (transparent tissue layer covering the surface), without open wounds (Figure 1A). In contrast, CED-affected fish weighing 0.1 g revealed open, irregular sores characterized by melanisation, which may be cause by macrophages, within the lesion and loss of mucus and scales in the affected areas (Figure 1B). In 30–900 g tilapia, lesions were visible to the naked eye (Figure 1C–G). The lesions appeared as open, non-melanotic sores located at the junction of the supraorbital, infraorbital, and otic cranial lateral line canals behind the eye. Some fish demonstrate a scar-like appearance surrounding the sores (Figure 1F). 

**Figure 2:**
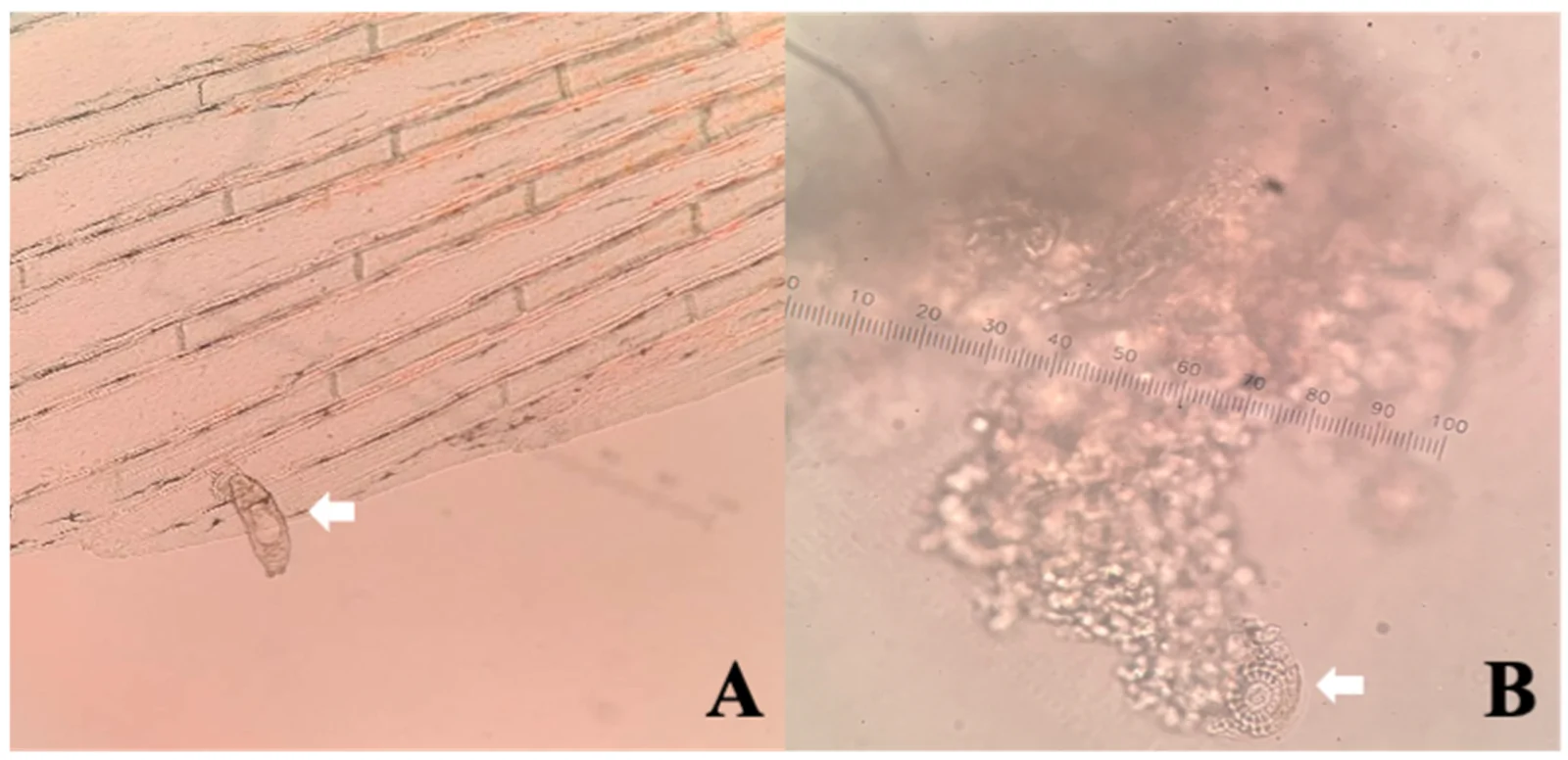
In fish weighing 0.1 g, the wet mount technique revealed ectoparasite infestation under a 10× light microscope, including a live Monogenean fluke from the tail fin (**A**) and *Trichodina* spp. from a deep skin scrape (**B**). The white arrow points to the parasite under the microscope.

During ectoparasite examination, a high level of infestation was observed in 0.1 g fish. In this group, CED lesion scrapings revealed remnants of ectoparasites, including monogenean flukes and *Trichodina* spp. (Figure 2), whereas larger fish (30–900 g) showed a lower incidence of infestation (Table 2). Bacterial culture and PCR assays confirmed the absence of both viral and bacterial infection in all examined fish.

### 3.2. Histopathology of the Affected Fish

**Figure 3:**
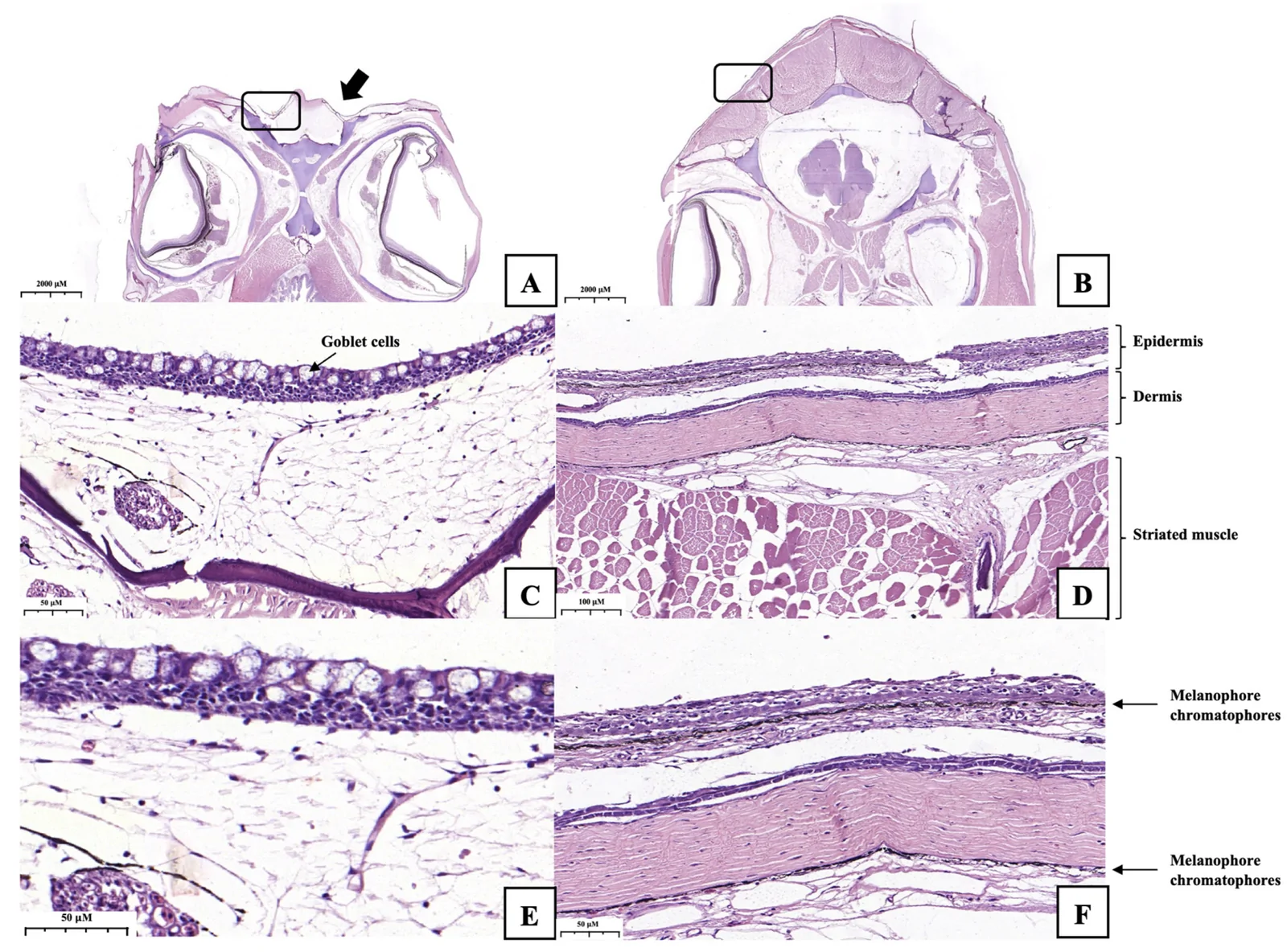
Histopathological analysis showed the absence of striated muscle in the ulcerative lesion of a 30 g fish (**A**), in contrast to the non-affected fish (**B**). The black arrow indicates the disappearance of striated muscle in the diseased fish. The black squares indicate the areas of higher magnification from (**A**–**D**). Panels (**C**) (20×) and (**E**) (40×) represent the affected fish, while panels (**D**) (10×) and (**F**) (20×) represent the non-affected fish. The affected fish (**C**,**E**) reveal an increased number of goblet cells in the epidermis and the disappearance of the dermis and melanophore chromatophores compared to the non-affected fish (**D**,**F**).

Histopathological analysis of the CED fish revealed the absence of the epidermis and dermis layers containing melanophore chromatophores and striated muscle at the lesion. The epidermis of the affected fish was newly formed, with epithelium and connective tissue that appeared as scar tissue. Furthermore, a remarkable increase in the number of mucus-secreting cells (goblet cells) was found in the epidermis of the affected fish (Figure 3). For non-CED fish, intact skin layers were observed, beginning with a stratified squamous epithelium containing Malpighian cells, with a thickness of three to five cell layers. Melanophore chromatophores were present in both the epidermal and dermal layers. The dermis consisted of dense and loose connective tissue, with striated muscle located beneath the dermal layer (Figure 3). 

### 3.3. Clinical Trials

The control group exhibited a significantly higher prevalence of ulcerative lesions compared to the treatment group across all fish sizes (*p* < 0.05). The incidence of ulcerative wounds (CED symptoms) was significantly higher in the control group, with 9, 12, and 8 of 30 fish affected at the first (0.02 g), second (0.05 g), and third (0.07 g) sampling points, respectively. In contrast, no skin ulcers were observed in the treatment group. The odds ratios were 25.71, 40.00, and 21.82 when comparing the control and treatment groups each week. In addition, the study’s total odds ratio was 71.31. Deep skin scraping revealed ectoparasite remnants in the ulcerative wounds of these fish, with the following findings: 1/9 in 0.02 g fish, 4/12 in 0.05 g fish, and 5/8 in 0.07 g fish. Moreover, wet mount technique from mucus scraping revealed that the 0.02 g fish in the control group had a notably higher level of ectoparasite infestation, with 19 out of 30 fish affected, compared to the other experimental groups (Table 3). 

## 4. Discussion

The pathological groups of chronic disorders affecting the lateral line canals are diverse. Nevertheless, the term CED is considered appropriate for this study based on the following key findings: the lesions are chronic, persisting in fish across a wide size range from 0.1 g to 900 g, with no associated morbidity or mortality. Furthermore, the lesion originates in the small canals of the lateral line and progresses to the area caudally to the eye. This specific anatomical involvement, which affects sensory structures present even in healthy fish, supports classifying this condition as a lateral line canal disorder. 

Previous studies across multiple fish species have reported similar gross CED pathology, typically characterized by focal erosion, ulceration, and epidermal loss around the lateral line canals of the head and trunk, often accompanied by fin erosion [16]. Likewise, we observed sores behind the eye extending to the cranial canal neuromasts, with pectoral fin erosion present from fry to grow-out. In contrast, histopathological features of CED vary among species, particularly in the epidermis overlying the lateral line canals [21]. Histopathological examination in our cases revealed no typical inflammatory responses, such as neutrophil or macrophage infiltration, suggesting that the injurious process may have already resolved or that the causative factor had been removed. An increased number of mucus-producing goblet cells was observed, likely reflecting a compensatory response to restore the disrupted skin barrier. These findings are consistent with reports of HIH in Nile tilapia [19] and CED in Murray cod [13], as well as studies in discus fish, channel catfish (*Ictalurus punctatus*), and sharpsnout seabream, in which the epidermis overlying the canals was reduced to a thin cell layer without associated inflammation [15,22,44].

Unlike previous reports that found no association between parasite infestation and CED-like lesions, the present study is the first to link ectoparasite infestation in Nile tilapia to CED-like lesions, beginning at the post-larval stage. Several risk factors have been associated with the development of CED-like lesions in various fish species, although the underlying cause remains hypothetical [20]. Previous studies reported that affected fish were often cultured at high stocking densities, resulting in increased stress, aggression, and deteriorated water quality [19]. Depigmentation of the cephalic lateral line in channel catfish was linked to chronic malnutrition or prolonged fasting [44]. Lesions in Murray cod were associated with groundwater exposure and resolved after transfer to river water, suggesting a role of heavy metal contamination [13]. Similarly, Meagre cultured in borehole water developed CED-like lesions, potentially due to elevated CO_2_ levels that promote osteoclast activity [20]. Comparable lesions were observed in ocean surgeons (*Acanthurus bahianus*) following the use of granulated activated carbon filtration [45]. Several pathogens have also been associated with CED-like conditions. A reovirus-like agent was isolated from the liver and spleen tissues of moribund marine angelfish (*Pomacanthus semicirculatus*) exhibiting lateral line depigmentation [46]. In addition, the hexamitid parasite *S. vortens* was isolated from ulcerative lesions and internal organs of diseased discus fish and angelfish [6].

Here, affected Nile tilapia (0.1–900 g) were maintained under acceptable water quality, and no systemic infectious diseases were detected. However, heavy ectoparasite infestation was consistently detected, with parasite remnants observed following deep skin scraping of ulcerative lesions in post-larval fish. These findings suggest a potential association between ectoparasite infestation and CED development. Therefore, subsequent clinical trials were conducted to evaluate this relationship in post-larval tilapia.

The trial began with 0.01 g of fish, corresponding to the stage at which fish are transferred from the hatchery to the nursery and experience initial exposure to a new environment. No CED lesions were observed in the ectoparasite-treated group. Odds ratio between treated and non-treated groups ranged from 21.82 to 40.00, indicating that ectoparasite reduction markedly lowered CED incidence. These findings suggest that ectoparasite load plays a critical role in lesion development. We propose that ectoparasites cause tissue damage that leads to ulcer formation, particularly in post-larval tilapia (~0.1 g; ~9 mm total length), which may be more susceptible due to an immature lateral line canal system. This is supported by Becker et al. [47], who reported that cranial canal neuromasts in cichlids begin forming at 5–8 mm and are not fully developed until 16–22 mm.

However, the observed reduction in lesions cannot be attributed exclusively to ectoparasite removal, as the treatment simultaneously targeted water quality. CED may originate from stress, predisposing fish to secondary ectoparasite infestations at lesion sites. Chlorine-based water treatment not only eliminated the ectoparasites but also reduced stress by improving water quality through microbial inactivation, oxidation of organic matter, and reduction in ammonia levels [48]. The importance of treatment strategy is further supported by Schultz et al. [49], who reported a reduction in the prevalence of ulcer lesions in juvenile Murray cod following disinfection with UV and electrolyzed water. Similarly, previous studies demonstrated that Murray cod and sharpsnout seabream developed lesions when reared in groundwater; however, these lesions resolved spontaneously when the fish were transferred to clean water [13,15]. Together, these findings suggest that effective CED prevention requires water treatment targeted not only at ectoparasite control, but also at improving overall water quality.

## 5. Conclusions

The present study described symptoms of chronic erosive dermatopathy (CED) in Nile tilapia beginning at the post-larval stage. No evidence for an active systemic infection was detected. Histopathology revealed neither acute nor chronic inflammatory responses but instead showed loss of several skin and underlying muscular layers. The only consistent finding across affected fish was ectoparasite infestation, detected by both wet mount examination and deep skin scraping of ulcerative lesions. Clinical trials demonstrated that water and tank treatment aimed at eradicating ectoparasites and improving water quality effectively reduced the incidence of CED in post-larval tilapia. Although the definitive cause of CED remains unconfirmed, this study provides the first evidence suggesting an association between ectoparasitic infestation and CED in Nile tilapia.

## Figures and Tables

**Table 1:** Primers used in this study.

Pathogens	Primers	Sequence	Size (bp)	Reference
Tilapia lake virus (TiLV)	*Nested ext-1*	TATGCAGTACTTTCCCTGCC	415	[35]
	*ME1*	GTTGGGCACAAGGCATCCTA		
	*7450/150R/ME2*	TATCACGTGCGTACTCGTTCAGT	250	
Tilapia parvo virus (TiPV)	*TiPV*	F: GAGATGGTGTGAAAATGAACGGG	NA	[36]
		R: CTATCTCCTCGTTGCTCGGTGTATC		
Universal 16S	*16S*	F: CCGAATTCGTCGAACAACAGAGTTTGATCCTGGCTC	1500	[37]
		R: CCCGGGATCCAAGCTTACGGCTACCTTGTTACGAC		
*Streptococcus* *agalactiae*	*F1*	F: GAGTTTGATCATGGCTCAG	220	[38]
	*IMOD*	R: ACCAACATGTGTTAATTACTC		
*Edwardsiella* spp.	*GyrB-1*	F: ATGTCAAATACGTATGACTCCT	1400	[39]
	*GyrB-1400*	R: ACCTCCTGCGAGGAGAGCAT		
*Aeromonas veronii*	*Act*	F: AGAAGGTGACCACCACCAAGAACA	323	[40]
		R: AACTGACATCGGCCTTGAACTC		
*Mycobacterium* spp.	*Sodlg*	F: GAAGGAATCTCGTGGCTGAATAC	540	[41]
		R: AGTCGGCCTTGACGTTCTTGTAC		

NA = not applicable.

**Table 2:** Summary of ectoparasite infestation results from both wet mount technique and deep skin scraping of lesions in all clinical cases.

Body Weight (g) (*n* = 15)	Skin Ulcer	Wet Mount Technique				Skin Scraping
		Mild	Moderate	Heavy	Not Found	
0.1	Negative	4	5	6	0	NA
0.1	Positive	1	1	13	0	14/15
30	Positive	0	0	0	15	NA
400	Positive	2	0	0	13	NA
900	Positive	0	0	1	14	NA

NA = not applicable.

**Table 3:** Summary of ectoparasite infestation results in clinical trials.

Sampling	Group (*n* = 30)	Skin Ulcer		Odds Ratio (OR)	Ectoparasite Detection	
		Positive	Negative		Wet Mount Technique	Deep Skin Scrape from Ulcer
1st Week (0.02 g)	Control	9	21	25.71 *	19/30 (63.33%)	1/9 (11.11%)
	Treatment	0	30		3/30 (10.00%)	NA
2nd Week (0.05 g)	Control	12	18	40.00 *	8/30 (26.67%)	4/12 (33.33%)
	Treatment	0	30		9/30 (30.00%)	NA
3rd Week (0.07 g)	Control	8	22	21.82 *	3/30 (10.00%)	5/8 (62.5%)
	Treatment	0	30		12/30 (40.00%)	NA
Total	Control	29	61	71.31 *	30/90 (33.33%)	10/29 (34.48%)
	Treatment	0	90		24/90 (26.67%)	NA

* = *p*-value < 0.05; NA = not applicable; Odds ratios were calculated using the Haldane–Anscombe correction (adding 0.5 to each cell) to accommodate zero counts in the treatment group.

## Data Availability

The original contributions presented in this study are included in the article. Further inquiries can be directed to the corresponding author.
